# Leakage-Controlled Classification of Dataset-Derived Ordinal p53-Signature Size Categories in Fallopian-Tube Immunohistochemistry

**DOI:** 10.3390/diagnostics16172869

**Published:** 2026-09-07

**Authors:** Ali Alhazmi

**Affiliations:** 1 Department of Computer Science, College of Engineering and Computer Science, Jazan University, Jazan 45142, Saudi Arabia; alihazmi@jazanu.edu.sa; Tel.: +966-56-827-8894; 2 Engineering and Technology Research Center, Jazan University, Jazan 82817, Saudi Arabia

**Keywords:** computational pathology, immunohistochemistry, ordinal classification, pathology foundation model, p53 signature, nested cross-validation

## Abstract

**Background and Objectives:** p53 signatures are segments of strongly p53-immunoreactive secretory epithelium in the fallopian tube. We tested whether fixed pathology-pretrained image representations could classify three ordered categories reconstructed from the public dataset’s reported cell-count intervals. **Methods:** The primary cohort comprised 113 canonical p53 20× fields from 67 patients: 59 Small (12–20 cells), 24 Medium (21–80), and 30 Large (>80). A 141-field partial-label cohort retained boundary-spanning intervals for sensitivity analysis. Twenty-six eligible feature/head pipelines were evaluated by three-repeat five-fold nested patient-grouped cross-validation. Within every outer fold, the pipeline was selected using inner validation only; primary performance metrics were computed within each repeat and averaged across the three repeats, while the resulting 339 held-out predictions were pooled only for the confusion-matrix visualization. Uncertainty was estimated using 2000 patient-cluster bootstrap replicates conditional on these recorded predictions. **Results:** The fold-wise nested selector achieved quadratic-weighted kappa (QWK) 0.687 (95% CI 0.547–0.785), macro-F1 0.628 (0.568–0.680), balanced accuracy 0.637 (0.581–0.693), macro-AUROC 0.805 (0.750–0.855), and macro-AUPRC 0.681 (0.633–0.754). Small, Medium, and Large sensitivities were 0.870 (0.801–0.928), 0.264 (0.159–0.386), and 0.778 (0.617–0.906); severe Small–Large errors occurred in 6.5% (2.9–10.6%). Equal patient weighting gave QWK 0.700. In a secondary locked UNI2-h/CORAL analysis, ordinal temperature scaling reduced ECE from 0.195 to 0.157 and negative log-likelihood from 1.291 to 0.778. **Conclusions:** Fixed representations provided useful internal discrimination of dataset-derived ordinal categories, with substantial fold variability and weak Medium-category sensitivity. The results do not validate lesion measurement, detection, diagnosis, or clinical use; independent pathologist-annotated evaluation is required.

## 1. Introduction

A p53 signature represents a continuous region of morphologically bland fallopian-tube epithelium containing at least 12 consecutive secretory-cell nuclei with strong p53 immunoreactivity [1]. It should not be confused with serous tubal intraepithelial carcinoma (STIC): the defining p53 staining can be present without the cytological atypia and high proliferative activity required for STIC. This distinction matters because an image-level model that estimates the extent of an already identified p53 signature is neither a cancer detector nor a patient-risk model.

The physical extent of a p53 signature is nevertheless a meaningful research phenotype. In the 250-subject retrospective cohort that formed the basis of the public image collection, Gibbard et al. categorized p53 lesions as Small (12–20 cells), Medium (21–80 cells), and Large (>80 cells) and related lesion-size distribution to oral-contraceptive exposure [2]. The present outcome is a computational fit to these dataset-derived ordinal categories; it is not an independently validated measurement of lesion size.

This setting creates methodological problems that can be easy to miss in a conventional image-classification pipeline. Several images may depict the same field at different focal planes; filename annotations may give an interval rather than an exact count; one patient can contribute multiple lesions; and the classes are ordered. If related views cross partitions, performance can be inflated. If a boundary-spanning interval such as 20–30 is forced into a single class, the reference standard becomes artificially certain. Finally, a Small-to-Large error is more consequential for an ordinal endpoint than a Small-to-Medium error, although nominal cross-entropy treats both as simply wrong.

The present study addresses these issues through a focused computational design: deterministic cohort reconstruction, patient- and relationship-aware nested validation, standardized fixed-feature comparison across ImageNet and pathology foundation encoders, ordinal learning, patient-cluster uncertainty, calibration, and acquisition-oriented robustness testing. The objective was to assess how reliably a compact fixed-feature framework could recover the ordered size phenotype in this p53-IHC collection and to describe the conditions under which performance changed.

The biological basis of this task is more focused than the broader literature on tubo-ovarian precursor detection. Folkins et al. operationalized p53 signatures as at least 12 consecutive strongly stained secretory nuclei [1]; Gibbard et al. later used the three ordered size bands adopted in this study [2]. Earlier digital IHC work showed that color deconvolution and cell segmentation can quantify stain burden and p53-positive structures [3,4], making a DAB-based proportional-odds model an appropriate task-specific baseline rather than relying only on generic neural networks.

Bogaerts et al. addressed a clinically different problem: detecting STIC/STIL in H&E whole-slide images. Their study included 323 STIC/STIL cases, 359 controls, and an external test set, and reported internal and external AUROCs of 0.98 and 0.95, respectively [5]. These results demonstrate the feasibility of AI for fallopian-tube pathology, but they should not be compared numerically with the present three-class p53-IHC size task, which uses preselected fields and interval-derived size labels rather than lesion-presence labels.

Large pretrained encoders have changed the practical trade-off for small datasets in computational pathology. UNI was developed as a general-purpose pathology representation [6], while CONCH introduced vision-language pretraining on more than one million histology image–caption pairs [7]. Virchow2 expanded self-supervision with mixed-magnification and was trained on a diverse corpus of histopathological slides [8]. Independent benchmarks caution against assuming that any single encoder is universally optimal. Campanella et al. compared public self-supervised pathology foundation models across clinical tasks [9], and Neidlinger et al. evaluated 19 models on 31 tasks from 13 cohorts, finding that relative performance varied with task and that advantages could narrow in low-data settings [10].

The stain domain is also relevant. Most widely used pathology foundation models were developed primarily from H&E-rich corpora, whereas p53 IHC encodes phenotype through both tissue morphology and chromogenic staining. StainNet, reported in 2026, was explicitly pretrained on more than 1.4 million patches from 20,231 IHC and special-stain whole-slide images, underscoring the emerging need for non-H&E-specific representation learning [11]. Recent ovarian-pathology studies likewise show that foundation-model rankings depend on endpoint and cohort [12,13]. These observations motivated a standardized fixed-feature comparison rather than selecting a foundation model a priori.

The size labels form an ordered response, Small<Medium<Large. CORAL models the outcome through rank-consistent cumulative thresholds [14], and CORN estimates ordinal probabilities through conditional tasks [15]. The literature supports pathology-pretrained transfer and ordinal modeling as relevant components, but it does not establish their behavior for these interval-derived p53-IHC categories and related focus variants. This task-specific gap motivated the present benchmark. A focused comparison with prior studies is provided in Table 1; the validation design is described in Section 2.

## 2. Materials and Methods

### 2.1. Study Overview

**Figure 1:**
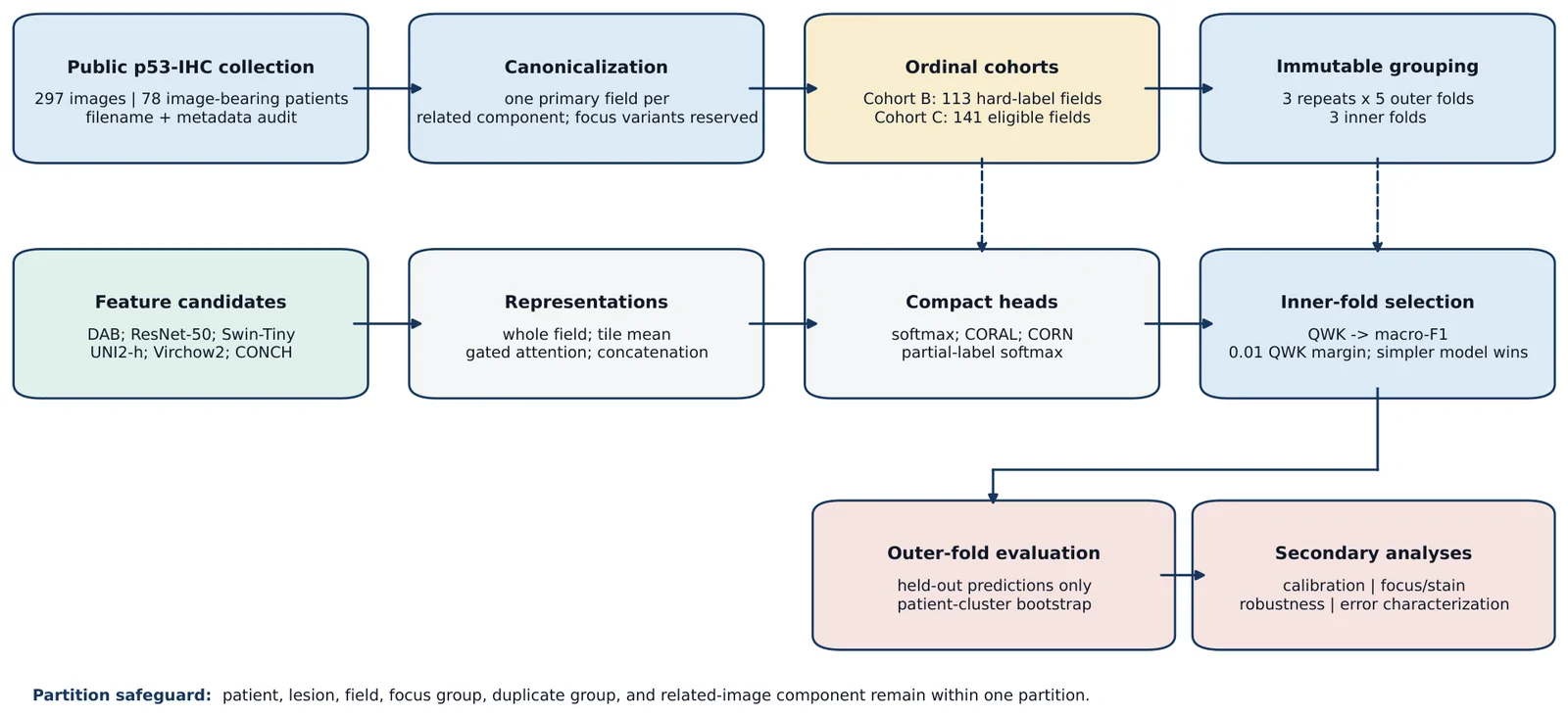
Leakage-controlled experimental workflow. Dataset reconciliation and canonical field construction precede immutable patient-grouped splitting. Encoder, representation, head, and regularization choices are selected within inner folds only; untouched outer-fold predictions are then used for performance estimation and error characterization.

Figure 1 summarizes the complete experimental pathway. The public TCIA release was first reconciled into biological patients and image-field relationships. Canonical p53 20× fields were then assigned either hard ordinal labels or permissible label sets. All candidate encoders were applied to immutable patient-grouped splits. Pipeline selection was repeated separately within every outer fold using inner-validation scores only; outer folds were used once for held-out estimation. Calibration and robustness were configuration-specific secondary analyses, and bootstrap inference was performed after the held-out predictions were locked.

### 2.2. Data Acquisition and Cohort Construction

Version 1 of the TP53-Precursor-Lesions collection was accessed through The Cancer Imaging Archive (TCIA), DOI 10.7937/WQGC-AB10, under CC BY 4.0 [16]. The package contained 297 histopathology images from 78 image-bearing biological patients and accompanying metadata. Images included p53, Ki-67, and H&E-associated views at multiple magnifications; the primary modeling target was restricted to verified canonical p53 20× fields. The source cohort comprised women older than 50 years who underwent benign gynecologic procedures, as described by the dataset authors. The released imaging metadata used for modeling did not provide a sex/gender variable suitable for within-cohort subgroup analysis, and no sex- or gender-derived variable was used as a predictor.

### 2.3. Source Immunohistochemistry and Image Acquisition

No tissue processing or immunohistochemistry was performed in the present computational study. The source investigators used formalin-fixed, paraffin-embedded fallopian-tube blocks and stained individual 4-μm sections for p53 according to Vancouver General Hospital clinical guidelines; the laboratory staining conditions are reported in Supplementary Table S1 of the source study [2]. Adjacent unstained sections from cases with large p53 lesions were stained for Ki-67 (MIB1) and H&E by the Molecular Anatomical Pathology Core at Vancouver General Hospital to support distinction of large p53 signatures from STILs/STICs [2]. Four gynecologic pathologists confirmed p53 signatures, with equivocal lesions undergoing senior consensus review, and source images were acquired using an Olympus BX46 microscope with an Olympus DP23 camera (Olympus Corporation, Tokyo, Japan) [2,16]. “Fixed” representation in this article refers only to pretrained encoder weights that were not updated during model fitting. It does not describe histological preparation; the source tissue was formalin-fixed and paraffin-embedded (FFPE).

### 2.4. Cohort Reconstruction

**Figure 2:**
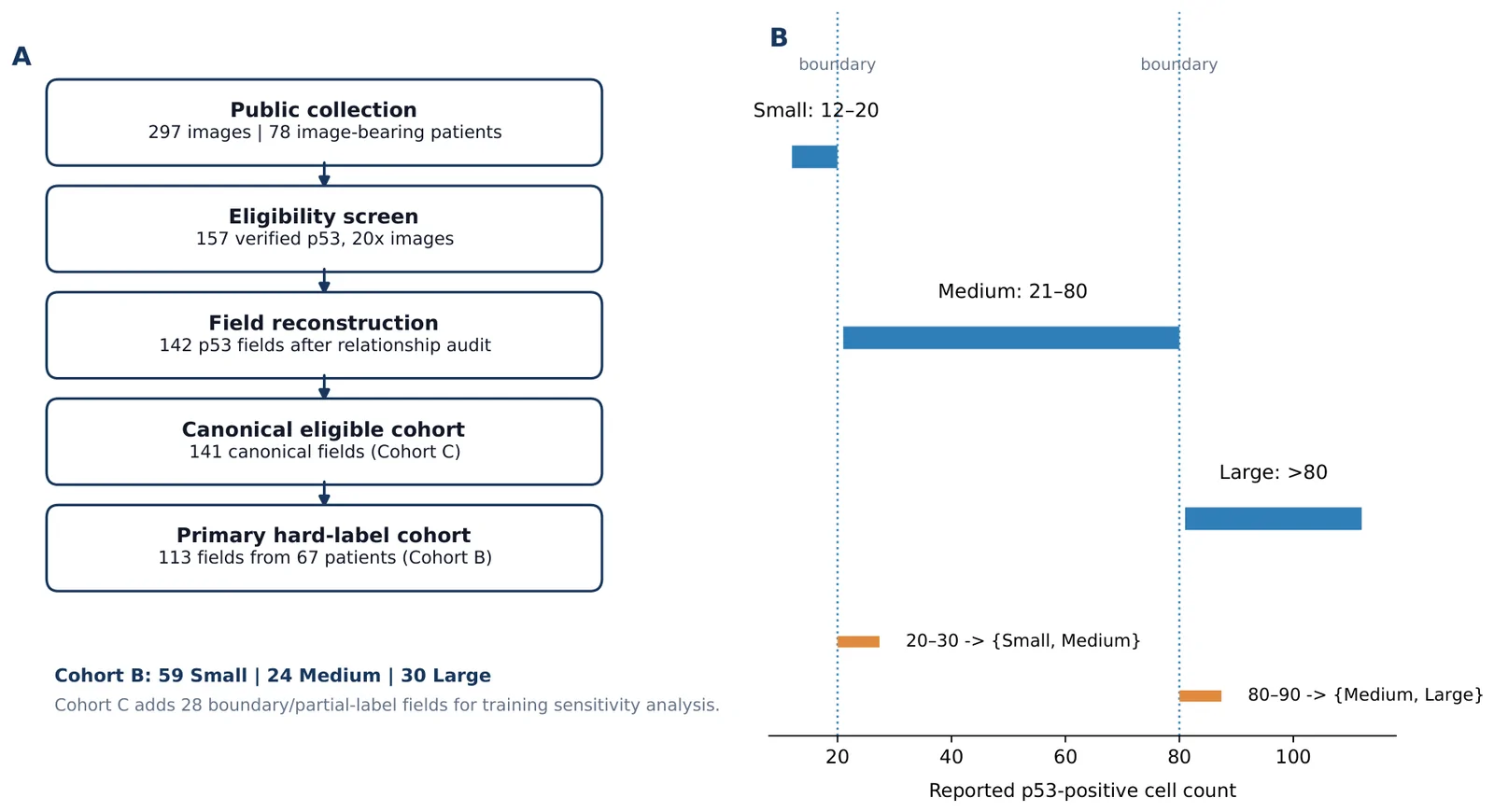
Cohort reconstruction and ordinal label definition. (**A**) Cohort reconstruction from the public collection to the primary hard-label cohort. (**B**) Ordinal label supports and boundary-spanning intervals. Cohort C contains 141 eligible canonical fields after relationship auditing; Cohort B contains the 113 fields whose complete reported count interval lies within a single ordinal category. Boundary-spanning intervals are retained as permissible adjacent-class sets for sensitivity analysis.

A deterministic parser aligned filename tokens with the metadata table and separated biological patient identifiers from specimen/slide suffixes. The 79 metadata rows contained one patient (OCP61) without a packaged image, reconciling the package to the official 78 image-bearing patients. Exact hashes, focus tokens, field identifiers, lesion groups, and connected related-image components were audited before splitting. Noncanonical focus views were excluded as independent primary observations and retained only for focus-consistency experiments. The audit yielded 157 p53 20× images, 142 reconstructed p53 fields, and 141 eligible canonical fields (Cohort C). Among them, 113 fields from 67 patients had unambiguous hard labels and formed Cohort B (Table 2 and Figure 2).

The locked class supports were defined as

(1) Y={0,1,2}={Small,Medium,Large},

with Small =[12,20], Medium =[21,80], and Large =[81,∞). A field received a hard label only when its complete reported interval was entirely contained within one category. Thus 20–30 remained {Small,Medium} and 80–90 remained {Medium,Large}. No midpoint assignment was used. For a partially labelled training field *i* with permissible set $S_{i}$, the interval-aware objective was

(2) $$L_{\mathrm{partial},i}=-\log\left(\sum_{k\in S_{i}}p_{ik}\right).$$

Partial-only fields were never used as hard-label validation or test examples.

### 2.5. Preprocessing

Each encoder used its official inference preprocessing, including required resizing/cropping and normalization. Whole-field representations were extracted from the complete canonical RGB field. For local representations, nonoverlapping 224×224 tiles were created at native resolution, edge tiles were white-padded, and tiles with at least 20% automated tissue were retained. If no tile passed the threshold, the most tissue-rich tile was retained and flagged. Up to 64 tiles were sampled at deterministic evenly spaced indices. Fold-local median imputation and feature standardization were fitted only on the corresponding training partition.

### 2.6. Augmentation and Perturbation Policy

No stochastic image augmentation was applied during fixed-feature extraction. This policy kept encoder comparisons deterministic and avoided introducing synthetic mixed labels for a count-defined ordinal endpoint. HED/DAB modifications, grayscale conversion, channel isolation, DAB removal, and Gaussian blur were reserved for post-lock robustness experiments and were never used to increase the effective training sample size.

### 2.7. Feature Extractors and Representations

A transparent baseline employed H–DAB color deconvolution [3] to summarize tissue fraction, DAB optical-density statistics, DAB-to-hematoxylin ratio, entropy, connected-component extent, and thresholded stain burden; a proportional-odds model produced ordered predictions.

Five fixed neural encoders were then benchmarked: ImageNet-pretrained ResNet-50 and Swin-Tiny, UNI2-h, Virchow2, and the CONCH visual tower (Table 3). All model revisions and checkpoint hashes were pinned, and all features were cached. The representation strategies included whole-field embeddings, mean-pooled tile embeddings,

(3) $${\bar{z}}_{i}=\frac{1}{m_{i}}\sum_{j=1}^{m_{i}}z_{ij},$$

gated-attention tile aggregation [17], and whole-plus-tile concatenation. Encoders were never updated.

**Figure 3:**
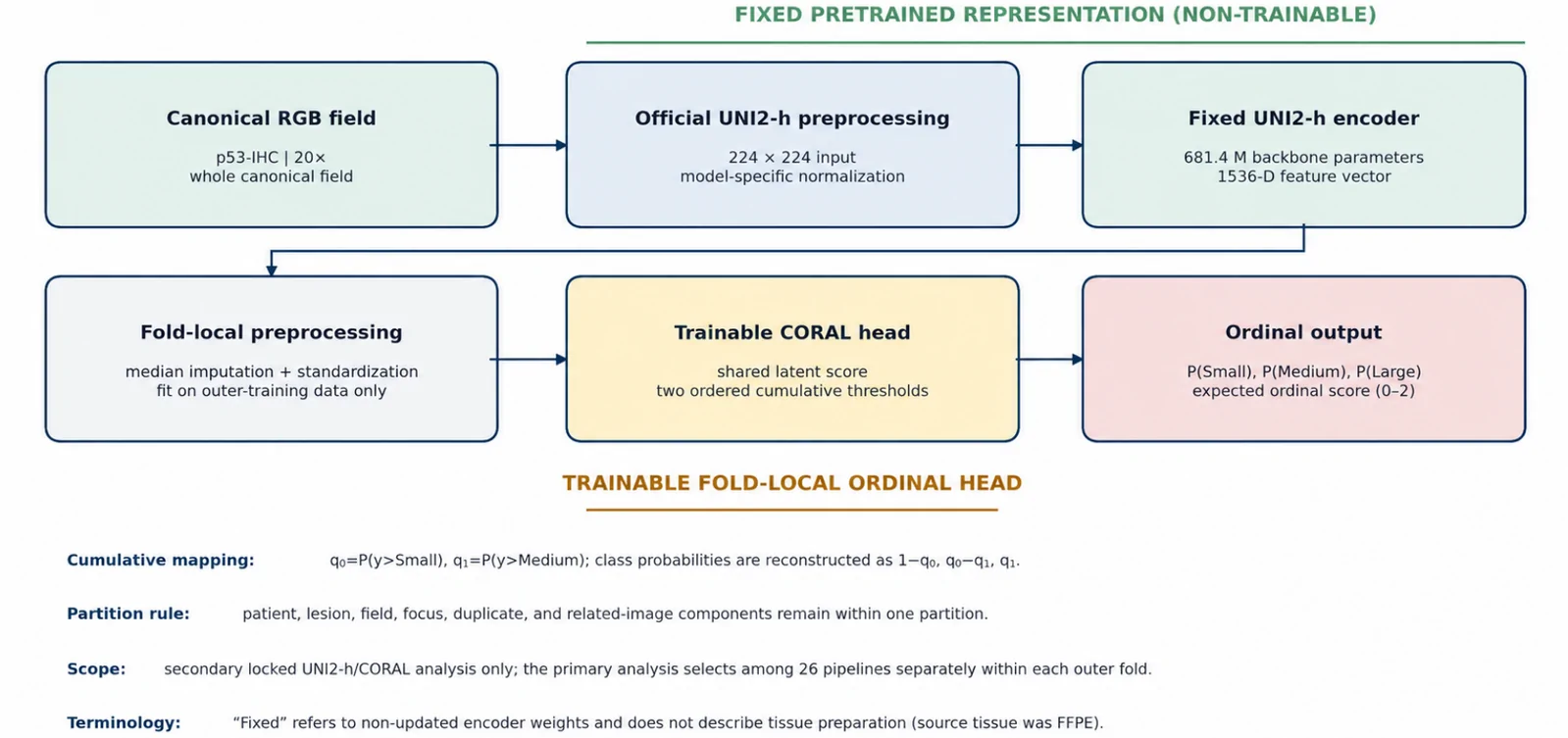
Locked UNI2-h/CORAL configuration used for secondary analyses. The pretrained UNI2-h encoder is non-trainable and produces a 1536-dimensional whole-field representation after official preprocessing. Median imputation and standardization are fitted only on the corresponding outer-training partition, and the compact CORAL head is trainable within that fold. Its two cumulative outputs, P(y>Small) and P(y>Medium), are converted into valid three-class probabilities. The figure also distinguishes this secondary locked configuration from the primary 26-pipeline fold-wise selector. “Fixed” refers to encoder weights and not to tissue preparation; the source tissue was formalin-fixed and paraffin-embedded.

The UNI2-h whole-field CORAL path used for the configuration-specific ordinal, calibration, robustness, and focus analyses is shown in Figure 3: a canonical RGB field is transformed with the official UNI2-h preprocessing, encoded by non-trainable pretrained weights into a fixed 1536-dimensional representation, standardized within the fold, and passed to a compact trainable CORAL head. This is a secondary locked configuration rather than the primary fold-wise selector reported below; here, “fixed” refers only to encoder weights that were not updated during model fitting.

### 2.8. Classifier and Ordinal Objectives

For image *i* with fixed *d*-dimensional feature vector $z_{i}\in R^{d}$, the nominal reference used

(4) $$p_{i}=\mathrm{softmax}\left(Wz_{i}+b\right),\;L_{\mathrm{CE}}=-\frac{1}{N}\sum_{i}\log p_{i,y_{i}}.$$

Here, W and b are trainable parameters, $p_{i}=\left(p_{i0},p_{i1},p_{i2}\right)$ is the three-class probability vector, $y_{i}\in \left\{0,1,2\right\}$ is the dataset-derived label, and *N* is the number of training fields. CORAL represented the three classes through two cumulative logits [14]:

(5) $$a_{ik}=w^{⊤}z_{i}-b_{k},\;q_{ik}=P\left(y_{i}>k|z_{i}\right)=\sigma \left(a_{ik}\right),\;k\in \left\{0,1\right\},$$

where $\sigma \left(x\right)={\left(1+e^{-x}\right)}^{-1}$, w is shared across thresholds, and ordered biases $b_{0}\leq b_{1}$ imply $q_{i0}\geq q_{i1}$. The uncalibrated class probabilities are $p_{i0}=1-q_{i0}$, $p_{i1}=q_{i0}-q_{i1}$, and $p_{i2}=q_{i1}$. CORN was evaluated as a conditional-probability ordinal alternative [15]. Compact neural heads used AdamW (learning rate 0.01), at most 500 epochs, and 30-epoch loss patience; the inner regularization grid was C∈{0.1,1,10} and dropout ∈{0,0.2}.

### 2.9. Repeated Nested Patient-Level Validation

We used three repeats of five outer folds, with three grouped inner folds per outer-training partition. The patient was indivisible, and automated checks additionally prohibited lesion, field, focus, duplicate, or connected related-image components from crossing any train/validation/test boundary. All 15 outer folds contained all three classes. For the interval-aware Cohort-C sensitivity path, the same outer patient assignment was enforced before inner splitting: a boundary/partial-label field was training-eligible only when its patient belonged to that fold’s outer-training patient set. Thus, partial-only fields associated with an outer-test patient were excluded from training, and partial-only fields were never used for outer validation or testing; the 15-fold patient-gate audit reports zero such admissions in every fold (Supplementary Data S2b). For the revised primary analysis, 26 complete, nonduplicated pipelines were eligible in each outer fold: the DAB proportional-odds baseline; whole-field and tile-mean ImageNet/pathology-encoder pipelines with nominal or ordinal heads; UNI2-h CORN and interval-aware variants; whole-plus-tile UNI2-h CORAL; and UNI2-h/Virchow2 gated-attention variants. Within each pipeline, hyperparameters had already been chosen and refitted using that outer fold’s training/inner-validation data only. Across pipelines, the highest mean inner QWK defined the reference; candidates within 0.01 were ordered by inner macro-F1, then lower representation complexity and a deterministic model identifier. The corresponding already-generated outer-test prediction was selected without consulting its label. This produced one selected pipeline per outer fold and 339 combined held-out predictions. The complete candidate scores, selected pipelines, hard-label patient/component manifest, and Cohort-C patient-gate audit are supplied as Supplementary Data S1–S3. Algorithm 1 summarizes the leakage-controlled nested evaluation and final prediction procedure.


**Algorithm 1** Leakage-controlled nested evaluation and final prediction generation.
**Require:** Canonical manifest *D*, patient/relationship groups *G*, outer splits *O*, inner splits *I*, encoder/head candidates C
  1:**for** each repeat *r* and outer fold *f* **do**  2:    Reserve outer-test patients $D_{test}^{\left(r,f\right)}$; never expose them to selection  3:    **for** each candidate c∈C **do**  4:        Fit preprocessing/head on each inner-training group and score inner validation by QWK and macro-F1  5:    **end for**  6:    Select $c^{*}$ by inner QWK, macro-F1, then lower complexity within the 0.01 QWK margin  7:    Refit $c^{*}$ on all outer-training patients using only locked settings  8:    Predict $D_{test}^{\left(r,f\right)}$ once; save class probabilities, ordinal score, split hash, and model provenance  9:
**end for**
10:Aggregate held-out predictions; compute patient-cluster CIs and paired differences; apply Holm correction within metric



### 2.10. Calibration, Robustness, and Statistical Inference

For the secondary locked UNI2-h/CORAL configuration, ordinal temperature scaling [18] used one positive scalar *T* for both cumulative logits in Equation (5):

(6) $$q_{ik}^{\left(T\right)}=\sigma \left(a_{ik}/T\right),\;k\in \left\{0,1\right\},\;T>0,$$

and reconstructed valid three-class probabilities as

(7) $$p_{i0}^{\left(T\right)}=1-q_{i0}^{\left(T\right)},\;p_{i1}^{\left(T\right)}=q_{i0}^{\left(T\right)}-q_{i1}^{\left(T\right)},\;p_{i2}^{\left(T\right)}=q_{i1}^{\left(T\right)}.$$

The same *T* was fitted separately in each outer fold by minimizing multiclass negative log-likelihood on inner out-of-fold predictions only. Positive shared scaling preserves $q_{i0}^{\left(T\right)}\geq q_{i1}^{\left(T\right)}$ and hence nonnegative probabilities summing to one; numerical clipping was used only at machine precision. No outer-test label was used to fit *T*. This calibration analysis was performed only for the secondary locked UNI2-h/CORAL configuration; calibration was not assessed for the primary fold-wise selector, which combines different selected pipelines across outer folds. Robustness analyses paired canonical fields with Gaussian blur, DAB intensity ±25%, grayscale, DAB-only, hematoxylin-only, DAB-removed, tissue-mask-only, and background-only variants. Thirty-nine real focus pairs were also evaluated, with no secondary views added to training.

Confidence intervals were estimated from 2000 valid patient-cluster bootstrap replicates, preserving all repeated observations of each sampled patient. These intervals are conditional on the recorded fold-wise selections and held-out predictions: the bootstrap resamples patients but does not repeat inner selection, feature extraction, or model refitting. Equal-patient-weight sensitivity estimates assigned each of the 67 patients total weight one before metric calculation. Eleven prespecified paired fixed-configuration differences were bootstrapped on identical sampled patients and adjusted within metric by Holm’s procedure [19]. Reporting was cross-checked against CLAIM 2024, TRIPOD + AI, and PROBAST + AI [20,21,22].

## 3. Results

All primary values come from the fold-wise pipeline selected using inner validation in each outer fold. Confidence intervals are patient-cluster intervals conditional on the recorded selections and held-out predictions unless stated otherwise.

### 3.1. Evaluation Metrics

QWK was the primary metric because it penalizes more distant ordinal disagreements more strongly. For K=3 classes, quadratic weights were

(8) $$w_{ij}=\frac{{\left(i-j\right)}^{2}}{{\left(K-1\right)}^{2}},\;\kappa_{w}=1-\frac{\sum_{ij}w_{ij}O_{ij}}{\sum_{ij}w_{ij}E_{ij}},$$

where *O* and *E* are observed and chance-expected confusion matrices [23]. We also reported macro precision, macro-F1, balanced accuracy (macro sensitivity), macro specificity, macro-AUROC, macro-AUPRC, mean absolute ordinal error

(9) $$\mathrm{MAOE}=\frac{1}{N}\sum_{i}\left|{\hat{y}}_{i}-y_{i}\right|,$$

and severe-error rate $N^{-1}\sum_{i}I(|{\hat{y}}_{i}-y_{i}\left|=2\right)$. Calibration used ECE, multiclass Brier score, and negative log-likelihood. Unless explicitly stated otherwise, repeated-validation performance estimates were calculated by computing each metric separately on the 113 held-out fields in each repeat and then averaging the three repeat-level values. Patient-cluster bootstrap intervals used the same repeat-averaged estimand. The pooled confusion-matrix display described later in the Results is intentionally different: it pools all 339 held-out predictions (113 fields × 3 repeats) to show integer confusion counts and row-normalized proportions. Therefore, nonlinear metrics such as precision and F1 recomputed directly from that pooled matrix need not equal the repeat-averaged per-class values reported later. Sensitivities coincide here because every repeat contains the same class supports.

### 3.2. Primary Fold-Wise Nested Selection

The fold-wise nested selector yielded repeat-averaged QWK 0.687 (95% CI 0.547–0.785) and macro-F1 0.628 (0.568–0.680), with balanced accuracy 0.637 (0.581–0.693), macro-AUROC 0.805 (0.750–0.855), macro-AUPRC 0.681 (0.633–0.754), MAOE 0.348 (0.264–0.439) classes, and a 6.5% (2.9–10.6%) severe Small–Large error rate (Table 4). The selected pipeline differed across folds: Virchow2 gated attention was chosen in 6/15 folds, UNI2-h whole-field CORAL in 2/15, and seven other pipelines in one fold each. Supplementary Table S5 reports all 15 choices and inner scores. Thus, the previously highlighted fixed UNI2-h/CORAL estimate (QWK 0.768, 0.656–0.858) is retained only as a configuration-specific secondary benchmark, not as the primary post-selection estimate.

**Table 4:** Primary performance of the fold-wise nested selector. Each metric was computed separately on the 113 held-out fields in each of the three repeats and then averaged across repeats; 95% patient-cluster bootstrap intervals target the same repeat-averaged estimand using 2000 valid replicates.

Analysis	QWK	Macro-F1	Balanced Accuracy	Macro-AUROC	Macro-AUPRC
Fold-wise nested selector	0.687	0.628	0.637	0.805	0.681
95% CI	0.547–0.785	0.568–0.680	0.581–0.693	0.750–0.855	0.633–0.754

**Figure 4:**
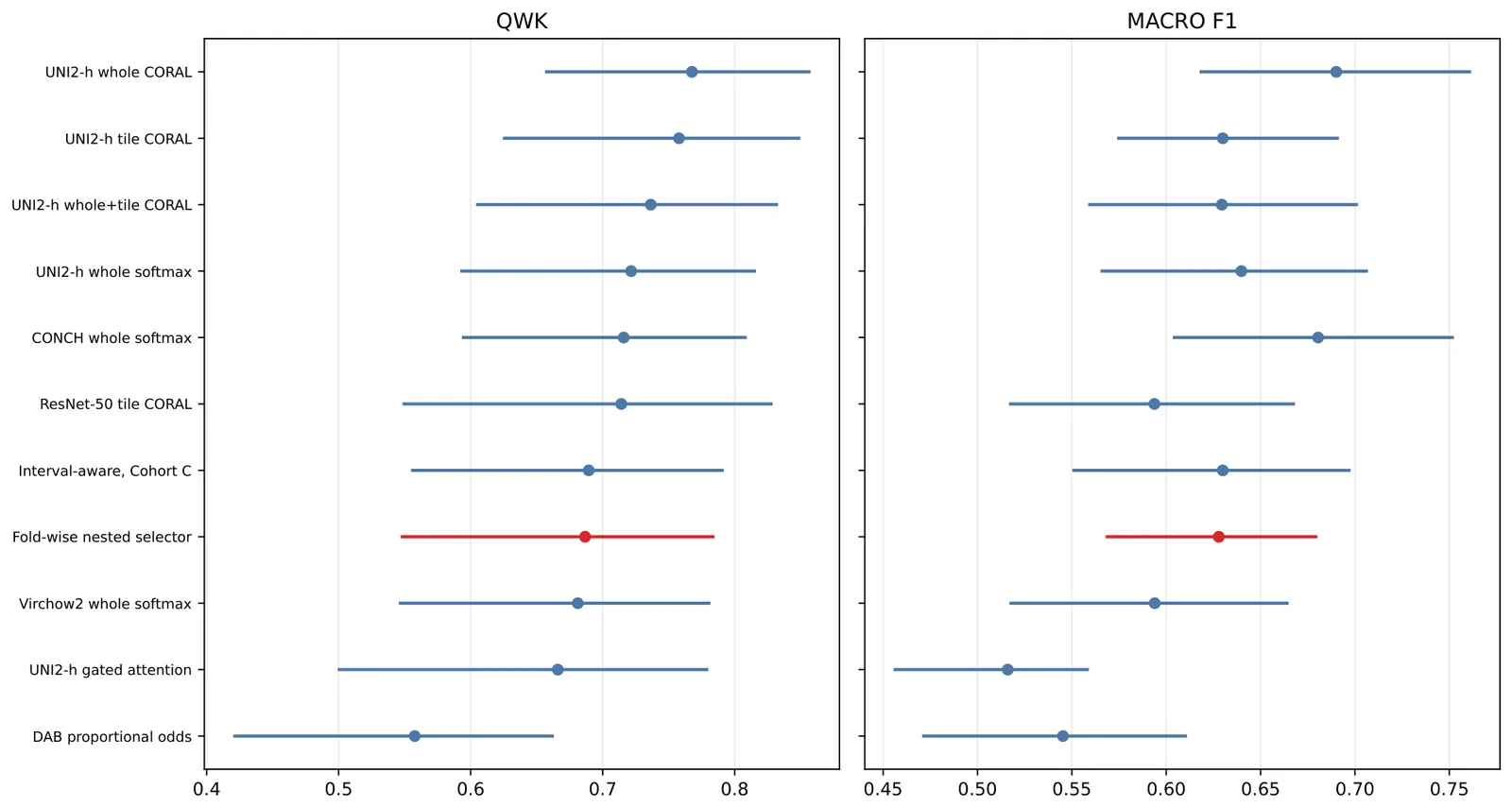
Held-out QWK and macro-F1 for the primary fold-wise nested selector and configuration-specific secondary benchmarks. Error bars show 95% patient-cluster bootstrap confidence intervals. Fixed-pipeline values must not be interpreted as the performance of a pipeline selected after reviewing this figure.

The selector chose one of 26 eligible pipelines separately in every outer fold using only inner-validation scores. Configuration-specific comparisons, including the previously highlighted fixed UNI2-h/CORAL result, are secondary and are shown in Figure 4, Table 5, and the Supplementary Materials.

**Table 5:** Focused ordinal and representation analysis using UNI2-h features. QWK and macro-F1 were computed separately within each repeat and averaged across the three repeats. These are fixed-configuration secondary analyses and are not pooled confusion-matrix estimates.

Configuration	QWK	Macro-F1
Whole softmax	0.722	0.640
Whole CORAL	0.768	0.690
Whole CORN	0.740	0.670
Interval-aware, Cohort C	0.689	0.630
Tile mean CORAL	0.758	0.630
Gated attention	0.666	0.516
Whole + tile CORAL	0.736	0.629

Fold-level QWK ranged from 0.386 to 0.918, macro-F1 from 0.426 to 0.796, and balanced accuracy from 0.472 to 0.800. Across repeats, QWK was 0.661, 0.625, and 0.774; corresponding macro-F1 values were 0.610, 0.567, and 0.706. These results show appreciable split sensitivity. Giving each patient equal total weight produced QWK 0.700, macro-F1 0.664, balanced accuracy 0.673, macro-AUROC 0.829, and macro-AUPRC 0.711 (Supplementary Tables S6 and S7).

### 3.3. Ordinal Objective and Representation Analysis

Among fixed secondary configurations, CORAL improved the UNI2-h whole-field point estimate compared with nominal softmax (QWK 0.768 vs. 0.722; macro-F1 0.690 vs. 0.640), but the paired QWK difference of +0.046 had a 95% CI of −0.004 to 0.114 and did not survive Holm correction. CORN remained intermediate (QWK 0.740; macro-F1 0.670). In Cohort C, the partial-label objective yielded QWK 0.689, with a paired QWK difference versus hard-label CORAL of −0.078 (95% CI −0.144 to −0.026; adjusted p=0.030). Under this protocol, retaining boundary-ambiguous fields through the partial-label objective did not improve on the high-certainty hard-label reference.

Increased representation complexity did not yield a consistent benefit among the fixed UNI2-h analyses. UNI2-h tile-mean CORAL reached QWK 0.758, whole-plus-tile fusion reached 0.736, and gated attention reached 0.666. Gated attention was lower than tile mean by −0.092 QWK (95% CI −0.173 to −0.032; adjusted p<0.001 at bootstrap resolution). Whole-plus-tile fusion showed no clear improvement over tile mean. These configuration-specific ordinal and representation ablations are summarized in Table 5.

### 3.4. Class-Wise Behavior and Ordinal Error

**Figure 5:**
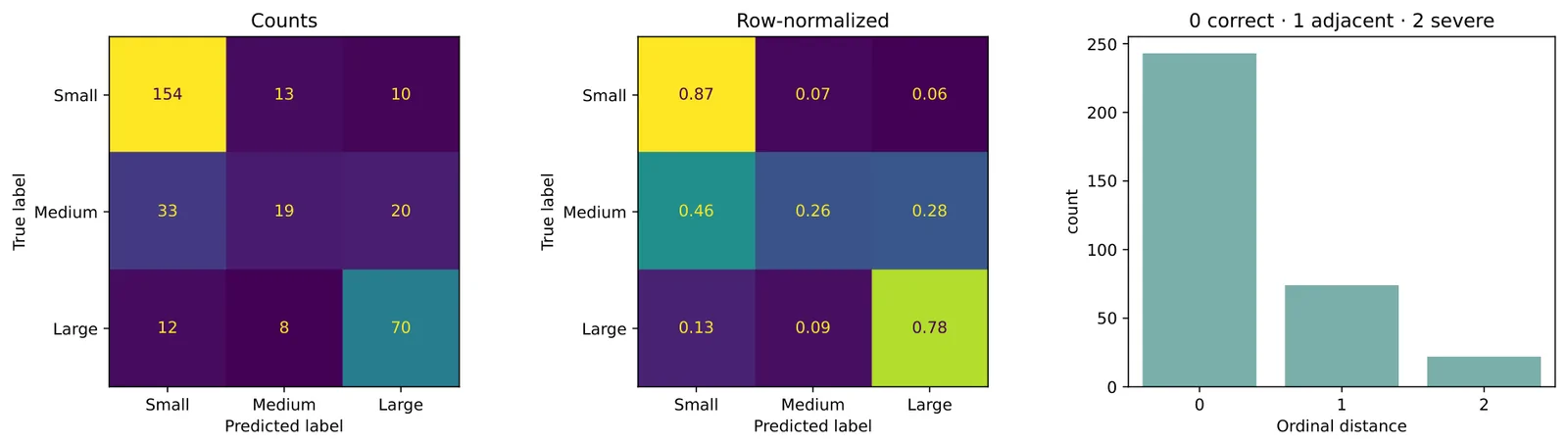
Pooled display of the fold-wise nested-selector confusion matrices and ordinal error distance over all 339 held-out predictions (113 fields × 3 repeats). Counts and row-normalized proportions are computed after pooling the three repeats for visualization; they are not the repeat-averaged metric estimates reported in Table 6. Severe errors correspond to a two-category Small–Large displacement.

Sensitivity was highest at the two ordinal extremes and lowest for the intermediate category (Table 6 and Figure 5). Small sensitivity was 0.870 (95% CI 0.801–0.928) and Large sensitivity was 0.778 (0.617–0.906), whereas Medium sensitivity was 0.264 (0.159–0.386). When the three repeats were pooled solely for the confusion-matrix display, the 339 selected held-out predictions contained 243 correct predictions, 74 adjacent-category errors, and 22 severe Small–Large errors. This pattern is consistent with an intermediate class that has fewer primary fields and is bounded on both sides by approximate count intervals.

### 3.5. Calibration, Focus Consistency, and Stain Perturbation

**Figure 6:**
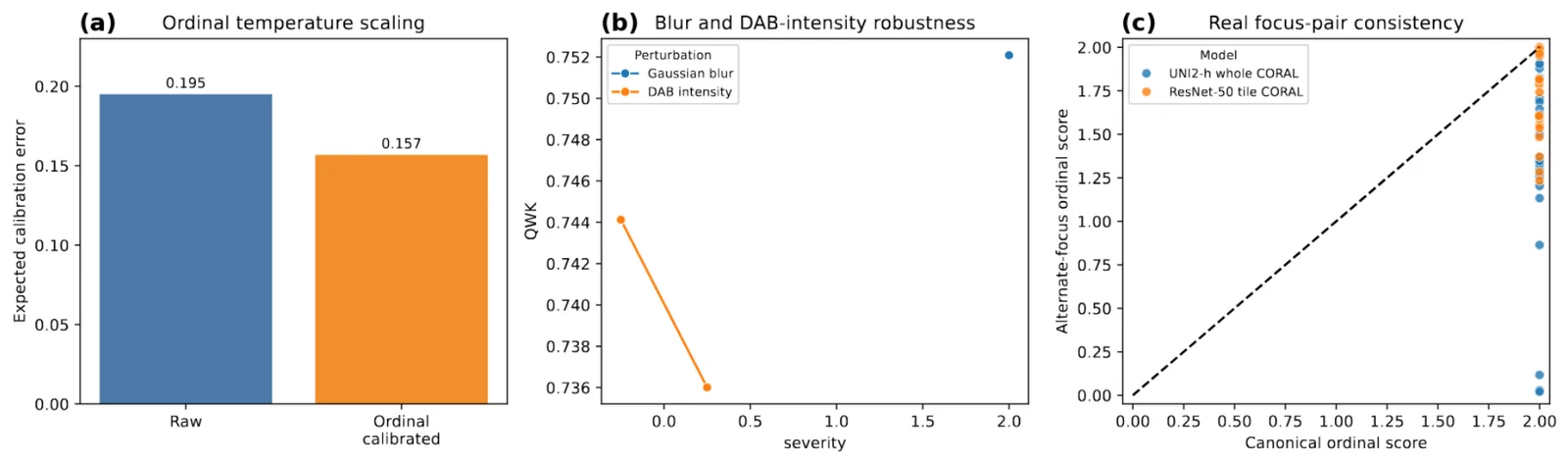
Configuration-specific secondary behavior. (**a**) Ordinal temperature scaling of the locked UNI2-h/CORAL model. (**b**) QWK under controlled test-time perturbations. (**c**) Real focus-pair predictions for the locked model and ImageNet comparator.

For the secondary locked UNI2-h/CORAL configuration, valid ordinal temperature scaling improved all three calibration summaries: ECE decreased from 0.195 to 0.157, Brier score from 0.391 to 0.385, and NLL from 1.291 to 0.778 (Table 7 and Figure 6). Calibration was not assessed for the primary fold-wise selector; the reported calibration results therefore apply only to this locked secondary configuration. Controlled blur caused only a modest QWK reduction, from approximately 0.766 to 0.752, while DAB −25% and +25% yielded 0.744 and 0.736. More extensive channel manipulations produced larger reductions: grayscale 0.713, DAB removed 0.611, hematoxylin-only 0.593, and DAB-only 0.435. Tissue-mask-only and background-only controls decreased to 0.029 and 0.042, respectively. This configuration-specific pattern is more consistent with reliance on combined morphology and chromogenic information than on a trivial mask/background shortcut.

Real focus variation changed predictions more than synthetic blur. Across 39 related focus pairs from only four patients, UNI2-h whole CORAL had 71.8% exact class agreement (95% patient-cluster CI 47.1–100.0%), a mean ordinal-score difference of 0.390 (0.047–0.693), and a mean Jensen–Shannon divergence of 0.160 (0.018–0.278). The ResNet-50 tile CORAL comparator showed 92.3% exact agreement (88.2–100.0%). The wide intervals preclude a general stability claim and identify prospective focus replication as a priority.

### 3.6. Qualitative Image Context and Interpretability Controls

**Figure 7:**
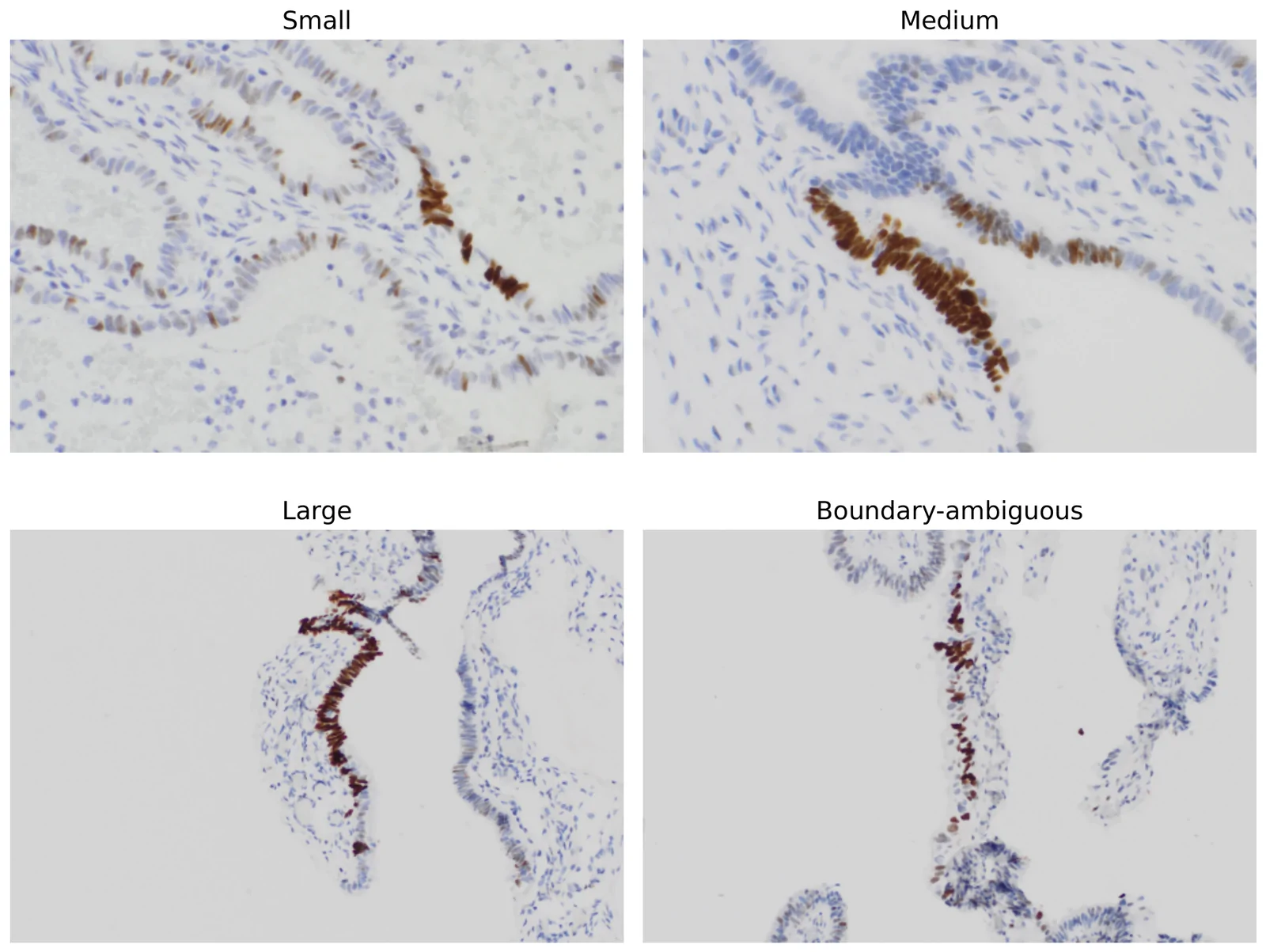
Representative p53-IHC fields from the locked cohort. The first three panels show hard-label Small, Medium, and Large fields; the fourth illustrates a boundary-ambiguous field. These images provide visual context and are not diagnostic exemplars or expert-defined ROIs.

Representative fields are shown in Figure 7. Interpretability was treated as a secondary sensitivity analysis and was not used to infer pathological reasoning. In the gated-attention analysis, attention had low overlap with the automated DAB proxy (mean attention mass in the proxy 0.0266; pointing-game hit rate 0), while removing DAB altered probabilities (mean $L_{1}$ change 0.313) and ordinal scores (mean absolute change 0.279). Because these maps cannot explain a heterogeneous fold-wise selector and the DAB mask is not a pathologist ROI, the findings are reported only as exploratory error characterization (Supplementary Figure S3).

### 3.7. Reproducibility and Experiment Traceability

The archived focused experiment package included 32 complete main prediction files with 339 rows each, 450 per-fold checkpoints, 480 structured run/selection records, and content-addressed feature caches. Before Revision 1, that source tree recorded 41/41 automated tests passed and 33/33 focused-protocol completion items. Revision 1 added machine-readable analysis outputs requiring 26 complete candidate pipelines, 15 outer-fold selection records, 339 unique held-out predictions, and zero patient/field/lesion/focus/duplicate/related-component crossings; all requirements were satisfied. The supplied supplement includes the analysis outputs, full outer-fold patient/component manifest, Cohort-C patient-gate audit, Revision 1 analysis source script, and a standalone package validator for the released tables. The original experiments ran with Python 3.12.13, PyTorch 2.12.0+cu130, torchvision 0.27.0+cu130, timm 1.0.16, scikit-learn 1.7.0, and scikit-image 0.25.2 on an NVIDIA RTX 2060 with 12,288 MiB VRAM. Fixed features were extracted with mixed precision and batches of one or two; model weights were pinned by revision and hash.

## 4. Discussion

The principal finding is that a selector restricted to inner-validation information provided useful but variable internal discrimination of the three dataset-derived categories. Its repeat-averaged QWK was 0.687 and macro-F1 was 0.628, with fold QWK ranging from 0.386 to 0.918. The difference between the strict fold-wise nested estimate and the previously highlighted fixed UNI2-h/CORAL value of 0.768 illustrates the distinction between a configuration-specific benchmark and a selection-aware primary estimate. The fixed results remain informative as secondary configuration benchmarks, while the fold-wise selector is the appropriate primary internal estimate.

The ordinal formulation is aligned with labels that discretize an underlying count continuum. CORAL’s fixed-configuration point-estimate gain over softmax is compatible with that structure, but the paired interval included zero. The evidence therefore supports ordinal modeling as a reasonable inductive bias without establishing a universal advantage for CORAL or any encoder. The variation in fold-wise selections likewise agrees with broader benchmarking evidence that pathology representation rankings are task- and sample-dependent [9,10].

The Medium category was the most challenging operating region. It contained 24 primary fields, sat between two decision boundaries, and yielded sensitivity 0.264 compared with 0.870 for Small and 0.778 for Large. The partial-label experiment tested whether retaining 28 additional ambiguous fields would help a fixed UNI2-h/CORAL configuration, but performance declined. A plausible interpretation is that the additional fields did not offset heterogeneous supervision near the thresholds; exact or consensus counts are needed to test that explanation.

Among fixed UNI2-h configurations, tile mean nearly matched whole-field QWK, while gated attention and whole-plus-tile fusion provided no consistent gain. In this cohort, adding a trainable aggregation mechanism may increase estimation variance without reliable benefit. That is a hypothesis rather than a demonstrated mechanism and does not conflict with gated-attention pipelines being selected in some individual folds.

The locked UNI2-h/CORAL robustness analyses provide narrower, configuration-specific evidence. Mild Gaussian blur and moderate DAB-intensity changes caused small QWK reductions, whereas DAB removal and channel isolation caused larger deterioration. Near-chance performance on background-only and tissue-mask-only controls argues against an obvious field-occupancy shortcut for that configuration. However, its real focus-pair agreement was 71.8%, with a very wide patient-cluster interval because all 39 pairs came from only four patients; synthetic blur did not capture all acquisition variation.

The interpretability analyses provide complementary, non-diagnostic evidence. The gated-attention ablation showed low spatial overlap with an automated DAB proxy, and those maps do not explain the heterogeneous fold-wise selector. Counterfactual DAB removal demonstrated sensitivity to stain information for a fixed model, but it does not establish use of the same morphological criteria as a gynecologic pathologist.

### 4.1. Strengths

The study reconciled biological patients and all identifiable field, lesion, focus, duplicate, and related-image relationships before splitting. Primary selection was performed separately in every outer fold; the full 15-fold choice table and patient/component manifest are released; uncertainty was clustered at the patient level; equal-patient and fold/repeat sensitivity analyses were added; and each reported estimate is traceable to a held-out prediction. The explicit conversion of cumulative CORAL outputs to valid class probabilities also makes the calibration procedure reproducible.

### 4.2. Limitations

This is a small, single public collection from one source context, with 113 hard-label fields from 67 patients and no independent test cohort. The categories were reconstructed from approximate filename count intervals rather than new blinded pathologist counts; consequently, the endpoint is dataset-derived classification, not validated lesion measurement. Although the revised selector uses no outer label within a fold, its 26-pipeline library was reconstructed from completed study artifacts, so broader analysis-development choices are not protected by an untouched external set. The patient bootstrap is conditional on recorded model selections and predictions and does not include selection/refitting variability. Calibration was not assessed for the heterogeneous primary fold-wise selector; the calibration analysis was limited to the locked UNI2-h/CORAL secondary configuration. Fold performance was heterogeneous; the Medium class was small, and the 39 focus pairs represented only four patients. Immunohistochemical staining was performed by the source investigators rather than within this computational study; the original laboratory conditions are documented in the source publication and its Supplementary Table S1 [2]. Most encoders were pretrained predominantly in H&E-rich domains. No new pathologist ROIs, reader study, subgroup fairness analysis, prospective assessment, or clinical-utility evaluation was performed. The task also starts after a p53-positive field has been selected; lesion detection, STIC/STIL diagnosis, screening, and patient-risk prediction are outside scope.

### 4.3. Future Directions

The next step is an independent laboratory/scanner cohort with prospectively specified rules, exact or consensus counts, enrichment of Medium and threshold-adjacent cases, and repeated focus acquisition across many patients. Pathologist ROI or nucleus-level annotation would permit comparison with human counting variability and biologically grounded localization. Nested refitting should be repeated in that cohort, followed only then by whole-slide lesion-localization and reader-assistance studies.

## 5. Conclusions

A leakage-controlled fold-wise nested selector achieved QWK 0.687 (95% CI 0.547–0.785) and macro-F1 0.628 (0.568–0.680) for three dataset-derived ordinal categories in preselected fallopian-tube p53-IHC fields. Medium sensitivity was 0.264, severe-error rate was 6.5%, and fold variability was substantial. The study is therefore a reproducible internal benchmark, not validation of automated lesion measurement or clinical use. Its patient-level grouping, fold-wise model selection, patient-weighted sensitivity analysis, ordinal calibration, and explicit component audits provide a transparent basis for subsequent external evaluation. Independent, pathologist-annotated, multi-site validation with prospectively specified counting and imaging procedures is the appropriate next step.

## Figures and Tables

**Table 1:** Selected literature most relevant to the present task. The table separates biological p53-signature work from computational pathology studies to avoid direct comparison of unlike endpoints.

Study	Data/Modality	Method or Endpoint	Relevance to This Study
Folkins et al. (2008) [1]	Fallopian-tube p53 IHC	p53 signature defined as ≥12 consecutive strongly p53-positive secretory nuclei	Establishes the microscopic lesion definition; does not define an AI task.
Gibbard et al. (2025) [2]	Retrospective cohort; p53 IHC	Lesions stratified as 12–20, 21–80, and >80 cells	Source of the ordinal size scheme and public image collection.
Bogaerts et al. (2024) [5]	H&E whole-slide fallopian tube; external test set	U-Net/ResNet50 STIC/STIL detection	Demonstrates AI for tubal precursor detection, but the endpoint, stain, annotations, and scale differ from p53-signature size estimation.
Chen et al. (2024) [6]	Large-scale histopathology	UNI foundation representation	Motivates domain-pretrained fixed representations for limited downstream data.
Lu et al. (2024) [7]	Histology image-text pairs	CONCH visual-language foundation model	Provides a complementary pathology representation evaluated here as a fixed visual encoder.
Campanella et al. (2025) [9]	Multiple clinical pathology cohorts	Benchmark of public self-supervised PFMs	Shows that foundation-model evaluation should be standardized across downstream tasks rather than assuming a universal winner.
Neidlinger et al. (2026) [10]	13 cohorts; 6818 patients; 9528 slides	19 foundation models on 31 weakly supervised tasks	Reports marked task dependence and diminished advantage of leading PFMs in some low-data settings.
Li et al. (2026) [11]	IHC and special stains	StainNet, stain-specific self-supervised PFMs	Highlights the current gap between mostly H&E-oriented pretraining and non-H&E downstream tasks such as p53 IHC.
Breen et al. (2025) [12]	Ovarian cancer histopathology	PFM benchmarking for subtype classification	Provides disease-area evidence that encoder rankings can vary with downstream ovarian-pathology tasks.

**Table 2:** Audited cohort construction and hard-label class support.

Item	Fields/Images	Patients
Physical collection	297	78
Verified p53, 20× images before field reconciliation	157	–
Reconstructed p53 fields	142	–
Cohort C: eligible canonical fields	141	–
Cohort B: hard-label canonical fields	113	67
Small (12–20 cells)	59	44
Medium (21–80 cells)	24	22
Large (>80 cells)	30	21

Patient counts by class are non-additive because one patient can contribute fields in more than one class. Cohort C adds 28 boundary/partial-label canonical fields for training sensitivity only; primary validation and testing use Cohort B.

**Table 3:** Fixed feature sources and the 26-pipeline candidate pool evaluated under identical patient-grouped outer splits. The final column gives the number of distinct pipeline configurations eligible in every outer fold; within-pipeline hyperparameter variants were resolved by inner validation.

Feature Source	Pretraining/Source	Backbone Parameters (M)	Feature Dimension	Input (px)	Pipelines (*n*)
H–DAB baseline	Color deconvolution	–	Handcrafted	Native	1
ResNet-50	ImageNet-1k	23.5	2048	224	4
Swin-Tiny	ImageNet-1k	27.5	768	224	4
UNI2-h	Pathology foundation	681.4	1536	224	8
Virchow2	Pathology foundation	631.2	2560	224	5
CONCH	Vision-language pathology	395.2	512	448	4
Total eligible pipelines					26

Candidate-pool composition: the H–DAB baseline contributes one proportional-odds pipeline. ResNet-50, Swin-Tiny, and CONCH each contribute whole-field and tile-mean representations with softmax and CORAL heads (4 each). UNI2-h includes those four core pipelines plus whole-field CORN, interval-aware whole-field, whole + tile CORAL, and gated attention (8 total). Virchow2 includes the four core pipelines plus gated attention (5 total). Backbone parameter counts refer to the feature extractors used for embedding generation rather than downstream classification heads. All neural encoder weights remained fixed. The 26-pipeline total matches Supplementary Data S3.

**Table 6:** Per-class performance of the fold-wise nested selector. Each class metric was computed separately within each repeat and then averaged across the three repeats; parentheses give 95% patient-cluster bootstrap intervals for the same repeat-averaged estimand.

Class	Precision	Sensitivity	Specificity	F1
Small	0.776 (0.685–0.863)	0.870 (0.801–0.928)	0.722 (0.607–0.825)	0.820 (0.757–0.873)
Medium	0.453 (0.268–0.635)	0.264 (0.159–0.386)	0.921 (0.890–0.949)	0.326 (0.203–0.446)
Large	0.701 (0.569–0.797)	0.778 (0.617–0.906)	0.880 (0.825–0.926)	0.737 (0.616–0.816)

Figure 5 instead pools all 339 held-out predictions to display integer counts and row-normalized proportions. Consequently, precision and F1 recomputed directly from that pooled confusion matrix can differ slightly from the repeat-averaged values reported here; sensitivity is numerically the same because each repeat contains identical class supports.

**Table 7:** Secondary locked UNI2-h/CORAL calibration before and after valid ordinal temperature scaling fitted on inner out-of-fold predictions. Calibration was not assessed for the heterogeneous primary fold-wise selector.

Metric	Raw	Calibrated
ECE	0.195	0.157
Brier score	0.391	0.385
Negative log-likelihood	1.291	0.778

## Data Availability

The source images and metadata are publicly available from The Cancer Imaging Archive (TCIA), DOI: https://doi.org/10.7937/WQGC-AB10. Derived nonidentifying experiment artifacts are available from the author upon reasonable request.

## Supplementary Materials

The following supporting information can be downloaded at https://www.mdpi.com/article/10.3390/diagnostics16172869/s1: Figures S1–S3, showing the interval-aware sensitivity analysis, per-class precision–recall curves, and exploratory gated-attention/DAB-proxy analysis; Tables S1–S15, documenting label mapping, split and leakage checks, fold-wise selection and performance, encoder provenance, calibration, focus consistency, interpretability, and the computing environment; and descriptions and provenance for Supplementary Data S1–S4.
