# Supplementary material for: Leakage-Controlled Classification of Dataset-Derived Ordinal p53-Signature Size Categories in Fallopian-Tube Immunohistochemistry
Source: Diagnostics (Basel). 2026 Sep 7;16(17):2869. doi: 10.3390/diagnostics16172869 (PMC13565410; doi:10.3390/diagnostics16172869)
Supplement: Supplementary file 1 [file diagnostics-16-02869-s001.zip › diagnostics-4515973-supplementary.pdf]

# Supplementary Material: Leakage-Controlled Classification of Dataset-Derived Ordinal p53-Signature Size Categories in Fallopian-Tube Immunohistochemistry

Ali Alhazmi

## 1. Scope and artifact hierarchy

This supplement records operational details and secondary analyses for the focused fixed-representation protocol. The definitive sources are the machine-readable manifest, immutable split files, held-out prediction tables, bootstrap tables, and trace JSON files in the experiment archive. A legacy end-to-end ResNet-50 exploratory run was superseded by the focused protocol, archived separately, and is not part of any reported result.

The accompanying `Revision1_Analysis` directory contains the following directly auditable files:

- Data S1: `outer_fold_patient_component_manifest.csv`;
- Data S2a: `outer_fold_counts_and_leakage_checks.csv`;
- Data S2b: `cohort_c_outer_test_patient_exclusion_audit.csv`;
- Data S3a: `candidate_pool_inner_scores.csv`;
- Data S3b: `outer_fold_selected_candidates.csv`;
- Data S3c: `foldwise_nested_selector_predictions.csv`;
- Data S4a: `ordinal_calibration_provenance.json`;
- Data S4b: `ordinal_calibration_fold_metrics.csv`;
- Data S4c: `ordinal_calibrated_predictions.csv`.

Additional fold/repeat, confidence-interval, equal-patient, focus, summary, analysis-source, and standalone package-validation files are included in the same directory.

## 2. Deterministic labels and canonicalization

Table S1: Locked count supports and examples of interval mapping.

| Count interval/token | Permissible class set | Primary hard label |
|---|---|---|
| 12–20 | {Small} | Small |
| 30–40 | {Medium} | Medium |
| 50–60 | {Medium} | Medium |
| 90–100 | {Large} | Large |
| 100–150 | {Large} | Large |
| 150+ | {Large} | Large |
| 20–30 | {Small, Medium} | None |
| 80–90 | {Medium, Large} | None |

Class supports were Small 12–20, Medium 21–80, and Large $\geq$ 81. For each interval $[l_i, u_i]$, the permissible class set contained every support interval with nonempty intersection. Open-ended intervals used $u_i = \infty$. A hard label was assigned only if the set had cardinality one.

Table S2: Project-level count-inheritance decisions encoded before splitting.

| Field | Locked decision |
|---|---|
| OCP131 | The images belong to one field. `OCP131_9_top_1_difFocus_count150+.tif` is the canonical primary p53 donor and is Large. The Ki-67 image is not a donor. The `possiblyseveral` and related views are robustness cases only. |
| OCP167 | Five `newFocus` images inherit 100–150. Only the explicitly counted donor is canonical; the inherited variants are focus-consistency cases. |

| Field | Locked decision |
|---|---|
| OCP190 | Five `difFocus` images inherit 100–150. Only the explicitly counted donor is canonical; the inherited variants are focus-consistency cases. |
| OCP243 | Two `difFocus` images inherit 50–60. Only the explicitly counted donor is canonical; the inherited variants are focus-consistency cases. |
| OCP246 | Two focus variants inherit 80–90. The canonical field retains {Medium, Large}, is excluded from hard-label Cohort B, and enters partial-label Cohort C. |

These decisions are technical metadata adjudications authorized by the Project Author/PI role. They are not represented as pathologist review.

## 3. Split construction and leakage checks

Table S3: Repeated nested validation contract.

| Item | Locked value |
|---|---|
| Outer design | 3 repeats $\times$ 5 folds |
| Inner design | 3 folds within each outer-training partition |
| Repeat seeds | 20260722, 3407, 8051 |
| Indivisible group | patient |
| Additional no-crossing keys | lesion, field, focus, duplicate, related component |
| Outer split files | 15 |
| Inner split files | 45 |
| Outer class requirement | Small, Medium, and Large present in every test fold |
| Selection | inner QWK; macro-F1; lower complexity within 0.01 QWK |
| Primary test cohort | Cohort B only |

The automated validator found zero patient, lesion, field, focus-group, duplicate-group, or connected-component overlap in every hard-label partition. Across each repeat, every one of the 113 Cohort-B fields appeared in exactly one outer test fold. Supplementary Data S1 (`Revision1_Analysis/outer_fold_patient_component_manifest.csv`) gives the patient and all component identifiers for every hard-label image in every outer train/test

partition (1,695 rows), and Supplementary Data S2a contains the corresponding fold counts and relationship-leakage checks. For the 28 boundary/partial-label fields added only in Cohort C, training eligibility was gated by the same outer patient assignment: a partial-only field was eligible only when its patient belonged to the outer-training patient set, before inner splitting. Supplementary Data S2b lists the outer-test patient IDs for all 15 folds and records zero partial-only fields from those patients admitted to training and zero partial-only fields used as outer validation/test cases in every fold.

Table S4: Outer-test patient and class counts. No partial-label or noncanonical focus/duplicate image occurred in any test partition.

| Repeat | Fold | Images | Patients | Small | Medium | Large |
|---|---|---|---|---|---|---|
| 0 | 0 | 23 | 15 | 12 | 5 | 6 |
| 0 | 1 | 22 | 12 | 12 | 4 | 6 |
| 0 | 2 | 23 | 12 | 12 | 5 | 6 |
| 0 | 3 | 22 | 14 | 11 | 5 | 6 |
| 0 | 4 | 23 | 14 | 12 | 5 | 6 |
| 1 | 0 | 23 | 15 | 12 | 5 | 6 |
| 1 | 1 | 23 | 14 | 12 | 5 | 6 |
| 1 | 2 | 22 | 13 | 12 | 4 | 6 |
| 1 | 3 | 23 | 14 | 12 | 5 | 6 |
| 1 | 4 | 22 | 11 | 11 | 5 | 6 |
| 2 | 0 | 22 | 13 | 12 | 4 | 6 |
| 2 | 1 | 22 | 13 | 11 | 5 | 6 |
| 2 | 2 | 23 | 12 | 12 | 5 | 6 |
| 2 | 3 | 23 | 15 | 12 | 5 | 6 |
| 2 | 4 | 23 | 14 | 12 | 5 | 6 |

For each of the 15 hard-label folds, the following diagnostic counts were all zero in both the train and test partition: partial/ambiguous images, noncanonical focus images, noncanonical duplicates, train–test patient overlap, and cross-partition patient, field, possible-lesion, lesion-group, focus-group, and duplicate-group identifiers. Patient counts in the complementary training partitions ranged from 52 to 56. The Cohort-C patient-gate audit separately verifies the training-only partial-label rule against each fold's explicit outer-test patient set; the count of partial-only fields from outer-test patients admitted to training is zero for all 15 folds.

Table S5: Pipeline selected in each outer fold using inner validation only.

| Repeat | Fold | Selected pipeline | Inner QWK | Inner F1 |
|---|---|---|---|---|
| 0 | 0 | UNI2-h tile mean softmax | 0.785 | 0.717 |
| 0 | 1 | ImageNet ResNet-50 tile mean CORAL | 0.816 | 0.584 |
| 0 | 2 | UNI2-h tile mean CORAL | 0.783 | 0.592 |
| 0 | 3 | Virchow2 whole-field softmax | 0.779 | 0.680 |
| 0 | 4 | UNI2-h whole-field softmax | 0.803 | 0.653 |
| 1 | 0 | Virchow2 gated attention | 0.782 | 0.736 |
| 1 | 1 | Virchow2 gated attention | 0.817 | 0.745 |
| 1 | 2 | Virchow2 gated attention | 0.789 | 0.608 |
| 1 | 3 | UNI2-h gated attention | 0.729 | 0.536 |
| 1 | 4 | ImageNet Swin-Tiny tile mean CORAL | 0.786 | 0.607 |
| 2 | 0 | UNI2-h whole-field CORAL | 0.798 | 0.676 |
| 2 | 1 | UNI2-h whole-field CORAL | 0.793 | 0.682 |
| 2 | 2 | Virchow2 gated attention | 0.781 | 0.783 |
| 2 | 3 | Virchow2 gated attention | 0.774 | 0.681 |
| 2 | 4 | Virchow2 gated attention | 0.845 | 0.769 |

The complete 26-pipeline score grid, within-pipeline hyperparameter identifier, selection log, prediction source, complexity rank, number of candidates within the 0.01-QWK margin, and explicit `outer_test_labels_used_for_selection=False` field are provided in Supplementary Data S3.

All intervals in Table ?? resample biological patients while preserving their repeated held-out observations. Within each bootstrap replicate, metrics are computed separately by repeat and then averaged across repeats. The intervals are conditional on the recorded

Table S6: Fold-level performance of the selected pipeline.

| Repeat | Fold | Patients | QWK | Macro-F1 | Bal. acc. | AUROC | AUPRC |
|---|---|---|---|---|---|---|---|
| 0 | 0 | 15 | 0.596 | 0.571 | 0.567 | 0.836 | 0.742 |
| 0 | 1 | 12 | 0.471 | 0.462 | 0.528 | 0.795 | 0.688 |
| 0 | 2 | 12 | 0.850 | 0.719 | 0.717 | 0.867 | 0.755 |
| 0 | 3 | 14 | 0.662 | 0.476 | 0.520 | 0.668 | 0.551 |
| 0 | 4 | 14 | 0.749 | 0.736 | 0.728 | 0.861 | 0.832 |
| 1 | 0 | 15 | 0.784 | 0.528 | 0.583 | 0.793 | 0.670 |
| 1 | 1 | 14 | 0.386 | 0.426 | 0.472 | 0.572 | 0.492 |
| 1 | 2 | 13 | 0.655 | 0.676 | 0.639 | 0.769 | 0.693 |
| 1 | 3 | 14 | 0.670 | 0.517 | 0.611 | 0.810 | 0.701 |
| 1 | 4 | 11 | 0.608 | 0.647 | 0.654 | 0.820 | 0.745 |
| 2 | 0 | 13 | 0.776 | 0.770 | 0.778 | 0.899 | 0.866 |
| 2 | 1 | 13 | 0.789 | 0.624 | 0.642 | 0.868 | 0.757 |
| 2 | 2 | 12 | 0.784 | 0.723 | 0.744 | 0.929 | 0.851 |
| 2 | 3 | 15 | 0.918 | 0.796 | 0.800 | 0.964 | 0.933 |
| 2 | 4 | 14 | 0.570 | 0.582 | 0.578 | 0.858 | 0.726 |

Table S7: Repeat-level variability and equal-patient sensitivity analysis for the fold-wise selector. Each Repeat row is calculated from that repeat's 113 held-out fields; the equal-patient row gives the separate patient-weighted sensitivity analysis.

| Analysis | QWK | F1 | Bal. acc. | AUROC | AUPRC | Sens. S | Sens. M | Sens. L | MAOE | Severe |
|---|---|---|---|---|---|---|---|---|---|---|
| Repeat 0 | 0.661 | 0.610 | 0.616 | 0.793 | 0.673 | 0.864 | 0.250 | 0.733 | 0.372 | 0.071 |
| Repeat 1 | 0.625 | 0.567 | 0.591 | 0.741 | 0.604 | 0.847 | 0.125 | 0.800 | 0.407 | 0.088 |
| Repeat 2 | 0.774 | 0.706 | 0.705 | 0.881 | 0.768 | 0.898 | 0.417 | 0.800 | 0.265 | 0.035 |
| Equal patient weight | 0.700 | 0.664 | 0.673 | 0.829 | 0.711 | 0.856 | 0.324 | 0.839 | 0.317 | 0.054 |

Table S8: Repeat-averaged fold-wise-selector performance with 95% patient-cluster confidence intervals. Each metric is computed within each repeat and then averaged across the three repeats; the bootstrap targets the same estimand.

| Metric | Estimate | 95% CI |
|---|---|---|
| QWK | 0.687 | 0.547–0.785 |
| Macro-F1 | 0.628 | 0.568–0.680 |
| Balanced accuracy | 0.637 | 0.581–0.693 |
| Macro-AUROC | 0.805 | 0.750–0.855 |
| Macro-AUPRC | 0.681 | 0.633–0.754 |
| Small sensitivity | 0.870 | 0.801–0.928 |
| Medium sensitivity | 0.264 | 0.159–0.386 |
| Large sensitivity | 0.778 | 0.617–0.906 |
| Mean absolute ordinal error | 0.348 | 0.264–0.439 |
| Severe error rate | 0.065 | 0.029–0.106 |

selections and predictions and do not include new inner selection, feature extraction, or model refitting within a bootstrap draw. The pooled 339-prediction confusion matrix in the main manuscript is a visualization and is not the estimand in Table ??.

## 4. Encoder and cache provenance

Table S9: Exact encoder sources and revisions.

| Encoder | Source | Exact identifier | Dim. |
|---|---|---|---|
| ResNet-50 | `timm/resnet50.a1_in1k` | State SHA-256 `0c33d05394c203e0...` | 2,048 |
| Swin-Tiny | `timm/swin_tiny_patch4_window7_224.ms_in1k` | State SHA-256 `a88cdf1532769363...` | 768 |
| UNI2-h | `MahmoodLab/UNI2-h` | Revision `d517a8dd47902dd7c308b3c36f63bce47e7b9a43` | 1,536 |
| Virchow2 | `paige-ai/Virchow2` | Revision `3158645804b69e3f3bc4439d4116edddf0840a72` | 2,560 |
| CONCH | `MahmoodLab/CONCH` | Weights `f9ca9f877171a28ade80228fb195ac5d79003357`; code `141cc09c7d4ff33d8eda562bd75169b457f71a62` | 512 |

Every encoder passed a synthetic input/output contract test before extraction. Ten final caches (five encoders $\times$ whole/tile) contained 141 canonical images per strategy and were keyed by the model source, exact revision, checkpoint hash, official transform, image hashes, strategy, tile settings, and cache schema. Partial per-image shards made extraction resumable; downstream code consumed only finalized caches.

## 5. Training algorithm

**Algorithm S1: Leakage-controlled nested fixed-feature evaluation**

1. Parse filenames and metadata; construct related-image components and canonical fields.
2. Map count intervals to hard or permissible label sets; lock Cohorts B and C.
3. Create immutable patient-grouped outer and inner splits and validate all relationship keys.
4. For each encoder and representation, verify the official transform/model contract and cache fixed embeddings.
5. For each outer fold:
   (a) fit imputation and scaling only in each inner-training partition;
   (b) fit candidate compact heads for each regularization/dropout value;
   (c) rank candidates by inner QWK, then macro-F1; apply the 0.01 lower-complexity tie-break;
   (d) refit the chosen configuration on the complete outer-training partition;
   (e) predict the untouched outer-test partition once and save probabilities, labels, groups, configuration, and hashes.
6. Pool held-out predictions by repeat; calculate metrics and 2,000-valid-replicate patient-cluster intervals.
7. Fit temperature scaling on inner out-of-fold logits only; apply it to paired outer predictions.
8. Run focus, perturbation, sensitivity, and secondary interpretability analyses without changing the cohort, splits, recorded selections, or held-out predictions.

## 6. Secondary fixed-configuration selection and comparisons

Whole field and tile mean fell within the 0.01 QWK tie margin in this aggregate summary. Because this aggregate table is configuration-specific, the revised main analysis repeats

Table S10: Aggregate inner-validation scores among fixed UNI2-h representations. This table is secondary and was not used to define the revised primary estimate.

| Representation | Mean inner QWK | Mean inner macro-F1 | Complexity rank |
|---|---|---|---|
| Whole-field CORAL | 0.7492 | 0.6180 | 0 |
| Tile-mean CORAL | 0.7463 | 0.6169 | 1 |
| Whole + tile CORAL | 0.7309 | 0.6022 | 2 |
| Gated attention | 0.7402 | 0.5416 | 3 |

selection within each outer fold (Table **??**).

## 7. Paired QWK comparisons

Table S11: Prespecified paired patient-bootstrap QWK contrasts among fixed configurations. Differences are model A minus model B.

| Model A | Model B | Difference (95% CI) | Holm $p$ |
|---|---|---|---|
| ImageNet ResNet tile CORAL | DAB proportional odds | +0.156 (+0.008, +0.306) | 0.312 |
| Virchow2 whole softmax | ImageNet ResNet tile CORAL | −0.033 (−0.124, +0.068) | 1.000 |
| UNI2-h whole CORAL | ImageNet ResNet tile CORAL | +0.054 (−0.016, +0.148) | 0.828 |
| CONCH whole softmax | ImageNet ResNet tile CORAL | +0.002 (−0.124, +0.137) | 1.000 |
| UNI2-h whole CORAL | Virchow2 whole softmax | +0.086 (+0.010, +0.178) | 0.261 |
| CONCH whole softmax | Virchow2 whole softmax | +0.035 (−0.066, +0.142) | 1.000 |
| CONCH whole softmax | UNI2-h whole CORAL | −0.052 (−0.167, +0.055) | 1.000 |
| UNI2-h CORAL | UNI2-h softmax | +0.046 (−0.004, +0.114) | 0.546 |
| Interval-aware Cohort C | Hard-label CORAL | −0.078 (−0.144, −0.026) | 0.030 |
| Gated attention | Tile mean | −0.092 (−0.173, −0.032) | $<$ 0.001[a] |
| Whole + tile | Tile mean | −0.021 (−0.086, +0.034) | 1.000 |

[a]Zero at 2,000-replicate bootstrap reporting resolution.

Only the lower interval-aware and gated-attention estimates remained supported after the reported within-metric Holm correction. An unadjusted interval excluding zero does not by itself authorize an encoder-superiority statement.

## 8. Calibration, focus, robustness, and interpretability

For the locked UNI2-h/CORAL secondary analysis, let $a_{i0}, a_{i1}$ denote the two ordered cumulative logits. A single positive fold-specific temperature was applied as $q_{ik}^{(T)} = \sigma(a_{ik}/T)$, followed by $p_{i0}^{(T)} = 1 - q_{i0}^{(T)}$, $p_{i1}^{(T)} = q_{i0}^{(T)} - q_{i1}^{(T)}$, and $p_{i2}^{(T)} = q_{i1}^{(T)}$. The temperature minimized three-class negative log-likelihood on inner out-of-fold predictions only. Supplementary Data S4 supplies all 15 temperatures, inner sample counts, and the explicit no-outer-label provenance record.

Table S12: Valid ordinal calibration for the secondary locked UNI2-h/CORAL configuration, averaged over outer folds and repeats.

| Metric | Raw | Temperature scaled | Change |
|---|---|---|---|
| Expected calibration error | 0.195 | 0.157 | −0.038 |
| Multiclass Brier score | 0.391 | 0.385 | −0.005 |
| Negative log-likelihood | 1.291 | 0.778 | −0.513 |

Table S13: Real focus-pair consistency with 95% patient-cluster intervals (39 canonical–alternate pairs from four biological patients per model).

| Model | Exact class agreement | Mean score difference | Mean JS divergence |
|---|---|---|---|
| ImageNet ResNet tile CORAL | 0.923 (0.882–1.000) | 0.148 (0.081–0.188) | 0.061 (0.032–0.077) |
| UNI2-h whole CORAL | 0.718 (0.471–1.000) | 0.390 (0.047–0.693) | 0.160 (0.018–0.278) |

Focus-pair QWK was degenerate because the paired subset had a narrow outcome distribution; agreement, score difference, and Jensen–Shannon divergence are the informative measures. The intervals resample the four patients and are correspondingly wide.

Table S14: Secondary interpretability values for 16 deterministically selected examples.

| Measure | Value |
|---|---|
| Mean gated-attention mass in DAB proxy | 0.0266 |
| Median gated-attention mass in DAB proxy | 0.0073 |
| Mean gated-attention mass in tissue | 0.6038 |
| DAB-proxy pointing hit rate | 0 |
| Mean probability-vector L1 change after DAB removal | 0.3133 |
| Mean absolute ordinal-score change after DAB removal | 0.2786 |

The attention values apply only to a gated-attention analysis and cannot explain the heterogeneous fold-wise selector. The DAB proxy is automated and was not reviewed as a lesion annotation.

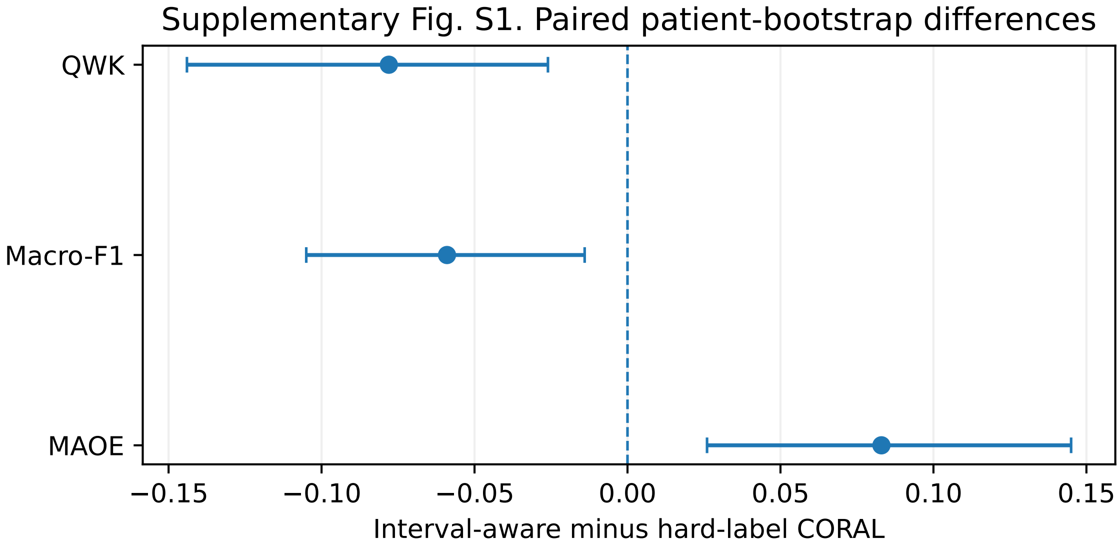


Figure S1: Hard-label CORAL versus interval-aware partial-label training. The partial-label model preserved permissible class sets in training; both models were evaluated on the same hard-label outer test fields.

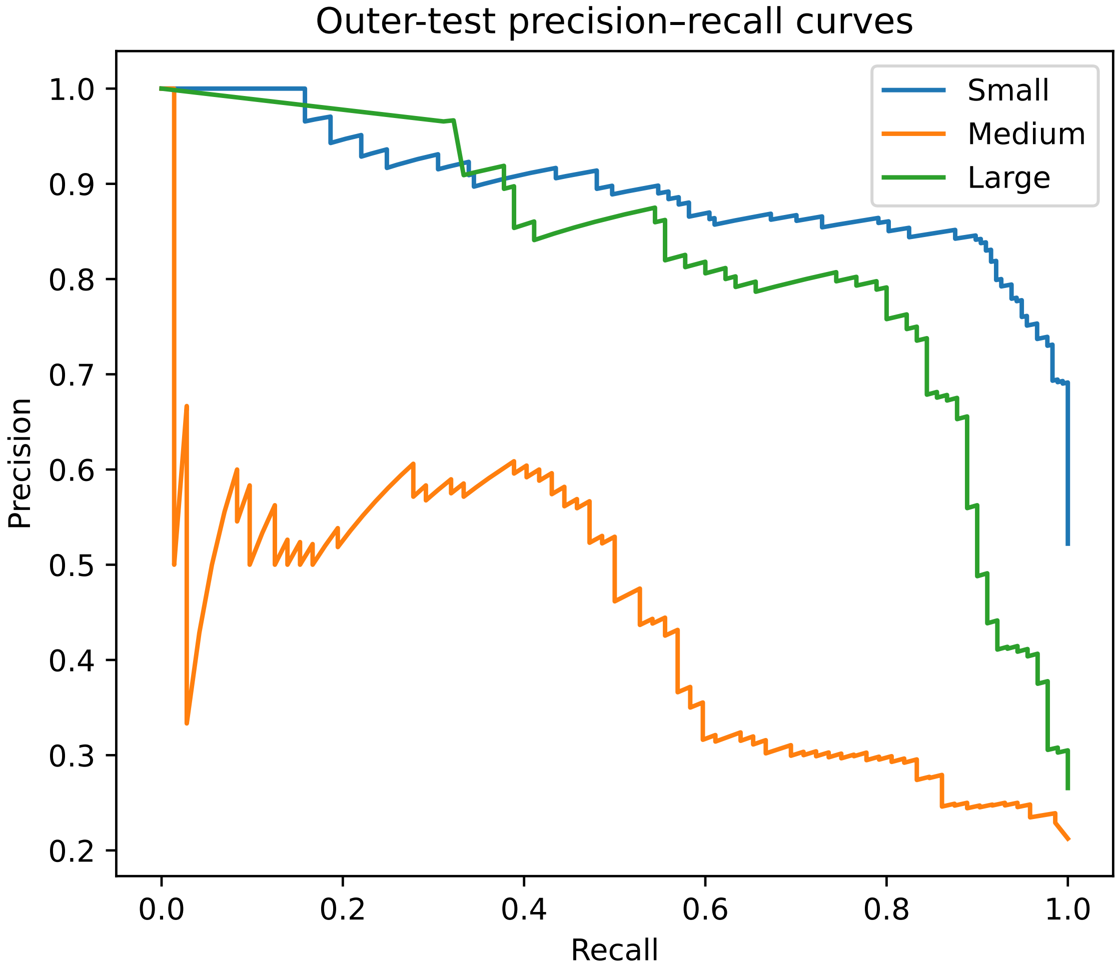


Figure S2: Per-class precision–recall curves for the principal fixed-feature configurations. Medium-class discrimination was consistently weaker than endpoint-class discrimination.

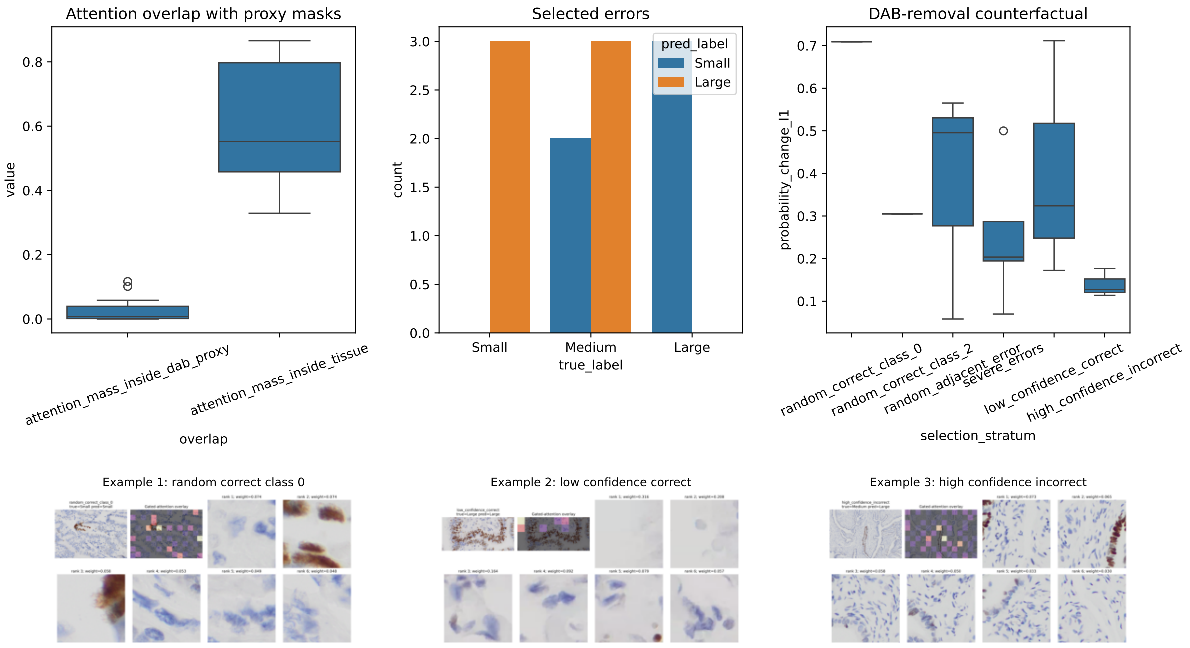


Figure S3: Exploratory gated-attention and DAB-proxy analysis. These maps belong to the nonselected gated-attention ablation and do not represent the final whole-field model or a pathologist annotation.

## 9. Reproducibility and completion record

Table S15: Audited computing environment.

| Component | Version/value |
|---|---|
| Platform | Linux 5.15, x86_64, glibc 2.39 |
| GPU | NVIDIA GeForce RTX 2060, 12,288 MiB |
| Python | 3.12.13 |
| PyTorch / torchvision | 2.12.0+cu130 / 0.27.0+cu130 |
| timm | 1.0.16 |
| NumPy / pandas | 2.2.6 / 2.3.0 |
| scikit-learn / scikit-image | 1.7.0 / 0.25.2 |
| statsmodels / Pillow | 0.14.4 / 11.2.1 |
| Archived pre-revision completion validator | 33/33 focused-protocol items |
| Archived pre-revision automated test suite | 41/41 passed |

The archived focused result package contains manifests, split files, cache provenance, prediction CSV files, checkpoint/log hashes, robustness outputs, bootstrap replicates/summaries, figures, tables, and validation reports. Revision 1 adds the analysis source `Revision1_Analysis/22_revision1_analysis.py`, its summary/provenance JSON files, the complete selection grid, fold/repeat/equal-patient tables, ordinal calibration outputs, focus intervals, the full hard-label patient/component manifest, and the Cohort-C patient-gate audit. The supplied `Revision1_Analysis/validate_revision1_package.py` is a standalone validator for the released machine-readable tables; the analysis source script is retained for provenance within the original archived experiment environment. The legacy validator is timestamp- and artifact-specific; its 33/33 value describes the archived focused protocol before the Revision 1 additions. Authentication tokens were not stored in any artifact.
